# Photo Protection of *Haematococcus pluvialis* Algae by Astaxanthin: Unique Properties of Astaxanthin Deduced by EPR, Optical and Electrochemical Studies

**DOI:** 10.3390/antiox6040080

**Published:** 2017-10-21

**Authors:** A. Ligia Focsan, Nikolay E. Polyakov, Lowell D. Kispert

**Affiliations:** 1 Department of Chemistry, Valdosta State University, Valdosta, GA 31698, USA; 2 Institute of Chemical Kinetics and Combustion, Novosibirsk 630090, Russia; polyakov@kinetics.nsc.ru; 3 Department of Chemistry, University of Alabama, Tuscaloosa, AL 35487, USA; lkispert@ua.edu

**Keywords:** astaxanthin, antioxidant, *Haematococcus pluvialis*, metal chelation, reactive oxygen species, photo protection, carotenoid, radical cation, proton loss neutral radicals

## Abstract

The antioxidant astaxanthin is known to accumulate in *Haematococcus pluvialis* algae under unfavorable environmental conditions for normal cell growth. The accumulated astaxanthin functions as a protective agent against oxidative stress damage, and tolerance to excessive reactive oxygen species (ROS) is greater in astaxanthin-rich cells. The detailed mechanisms of protection have remained elusive, however, our Electron Paramagnetic Resonance (EPR), optical and electrochemical studies on carotenoids suggest that astaxanthin’s efficiency as a protective agent could be related to its ability to form chelate complexes with metals and to be esterified, its inability to aggregate in the ester form, its high oxidation potential and the ability to form proton loss neutral radicals under high illumination in the presence of metal ions. The neutral radical species formed by deprotonation of the radical cations can be very effective quenchers of the excited states of chlorophyll under high irradiation.

## 1. Introduction 

**Figure 1:**
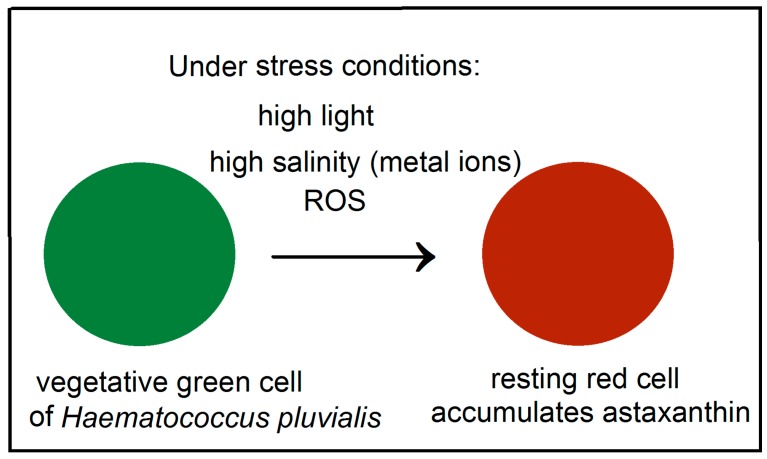
Schematic diagram of astaxanthin’s accumulation in H. pluvialis alga under stress conditions. The high amount of astaxanthin present in the resting cells is rapidly accumulated when the environmental conditions become unfavorable for normal cell growth.

Astaxanthin, belonging to the xanthophyll class of carotenoids, is well known for its unusual high antioxidant activity. Astaxanthin accumulates in *Haematococcus pluvialis* green microalga in open pond systems such as natural waters (lakes, lagoons, ponds) and artificial ponds specially designed for cultivation of alga (raceway ponds). *Haematococcus pluvialis* is the richest source of natural astaxanthin and is used to feed livestock and fish, and as a source of biofuel. For example, Kauai, Hawaii has one of the largest algae biofuel production facilities in the United States. The massive astaxanthin accumulation in *Haematococcus pluvialis* (Figure 1) is a cellular response to stress conditions such as bright light, high salinity, high carbon/nitrogen ratio and low availability of nutrients (Figure 1). Astaxanthin is known to play a protective role in response to these environmental stress conditions which would be deleterious to many other microalgae. Astaxanthin production can be induced by low nitrate or phosphate, high temperature or light, or the addition of sodium chloride in the culture medium [1,2,3]. 

Ferrous ion Fe^2+^, which sustains Fenton chemistry and reactive oxygen species (ROS) such as singlet oxygen (^1^O_2_), superoxide radical anion (O${}_{2}^{•-}$), hydrogen peroxide H_2_O_2_, peroxy radical (RO${}_{2}^{•}$), were also shown to enhance astaxanthin production of *Haematococcus pluvialis* [1,2,3]. Photosynthetic algae can generate these ROS through chloroplast photosynthesis and mitochondrial respiration under stress conditions. They can be used as signal molecules to initiate production and accumulation of astaxanthin [1]. The green vegetative cells consist of primary carotenoids such as lutein (75–80%) and β-carotene (10–20%), violaxanthin, neoxanthin, and zeaxanthin, as well as chlorophyll a and b [3]. In the red cells, the primary carotenoids are replaced by secondary carotenoids, mainly astaxanthin (80–99% of total carotenoids), while the ratio of carotenoids to chlorophylls increases by an order of magnitude [3]. The majority of astaxanthin is not deposited in its free form but it exists within the cell as fatty acid esters of astaxanthin, usually monoesters or diesters of palmitic, oleic, or linoleic acids. This type of modification is required for the deposition of this highly polar molecule within non-polar matrix of lipid droplets [3]. Approximately 70% monoesters, 25% diesters, and only 5% of the free astaxanthin is present in the red cells of *H. pluvialis* [3]. The typical composition of *Haematococcus algae* biomass consists of common carotenoids, lipids, proteins, carbohydrates, and minerals [3,4]. Under stress, *Haematococcus pluvialis* accumulates 1% of cell mass as carotenoids, 1% iron, 1% magnesium, and 2% calcium [4]. According to Lorentz [4] the 1% cell mass carotenoid accumulated under stress contains about 70% monoesters of astaxanthin, 10% diesters of astaxanthin, 5% free astaxanthin, the remaining 15% consisting of a mixture of β-carotene, canthaxanthin, lutein and other carotenoids. 

Excess light energy accelerates the generation of ROS. In order to escape from exposure to excess light, most plants have evolved various defense mechanisms to prevent the generation of ROS, such as non-photochemical quenching (NPQ) of chlorophyll fluorescence, plastid terminal oxidase (PTOX), Photosystem I (PSI) cyclic electron transport and the functioning of antioxidant enzymes [5]. For example, NPQ, of which the main component is the energy-dependent quenching (qE), can dissipate excess absorbed light energy in photosystem II (PSII). The qE mechanism in PSII is a pH-dependent response that enables plants to regulate light harvesting in response to rapid fluctuations in light intensity and is controlled by the xanthophyll cycle that under dark converts zeaxanthin into violaxanthin [6]. During cell transformation and astaxanthin accumulation in *H. pluvialis*, the photoprotective capacity NPQ decreases gradually [5]. However, it has been suggested that astaxanthin can protect *H. pluvialis* cells against oxidative stress through two distinct antioxidative mechanisms, the defensive enzyme system and the astaxanthin itself, astaxanthin being more efficient than the defensive enzyme system [5]. Astaxanthin reacts with ROS much faster than the protective enzymes, and has the strongest antioxidative capacity to protect against lipid peroxidation [7]. While in green vegetative cells, astaxanthin is very low or absent and scavenging of ROS is inevitably reliant on antioxidative enzymes, in red cells, astaxanthin quenches O${}_{2}^{•-}$ before the protective enzymes could act. It was shown [5] that in *H. pluvialis* grown outdoors, NPQ, PTOX, CEF-I (cyclic electron flow around PSI), defensive enzymes activities and the accumulation of amounts of astaxanthin can protect cells against photoinhibition. Tolerance to excessive ROS is greater in astaxanthin rich cells and the accumulated astaxanthin in red cells can function as a protective agent against oxidative stress damage [8].

## 2. Unique Properties of Astaxanthin

So why is astaxanthin so successful when exposed to stress conditions such as high light, high salinity, metal ions and ROS? Unique properties of astaxanthin detected in our EPR (electron paramagnetic resonance), optical and electrochemical studies such as the ability to form chelate complexes with metals, the ability to esterify with fatty acids to form mono and diesters, large oxidation potentials for astaxanthin and its esters, the inability to aggregate in the ester form, and the ability to form proton loss neutral radicals under high illumination explain its efficiency and might reveal photo protection mechanisms that have not been considered before. 

### 2.1. The Ability of Astaxanthin to Form Chelate Complexes with Metals 

Astaxanthin has unique properties based on its molecular structure. The presence of the hydroxyl (–OH) and keto (C=O) groups at position(s) C3(C3’) and C4(C4’) symmetrically on both cyclohexene rings can explain the ability to coordinate with metals. The astaxanthin cyclohexene ring has a structure similar to many α-hydroxy-ketones and hydroxy quinones which possess high biological activity, including the ability to form chelate complexes with metal ions. 

How is metal chelation at carbonyl and hydroxyl groups important for astaxanthin’s protection function? We have found [9] that some chelators can completely inhibit the ROS production induced by Fe ions in both dark and photoinduced processes. It was shown [9] that chelating of Fe^2+^ or Cu^2+^ ions can completely inhibit the production of ^•^OH and ^•^OOH radicals formed in photo and dark Fenton reactions. In addition, chelate complexes often have additional bands in the absorption spectrum at longer wavelength [10,11]. This might be important for the light scavenging ability of astaxanthin.

**Scheme 1:**
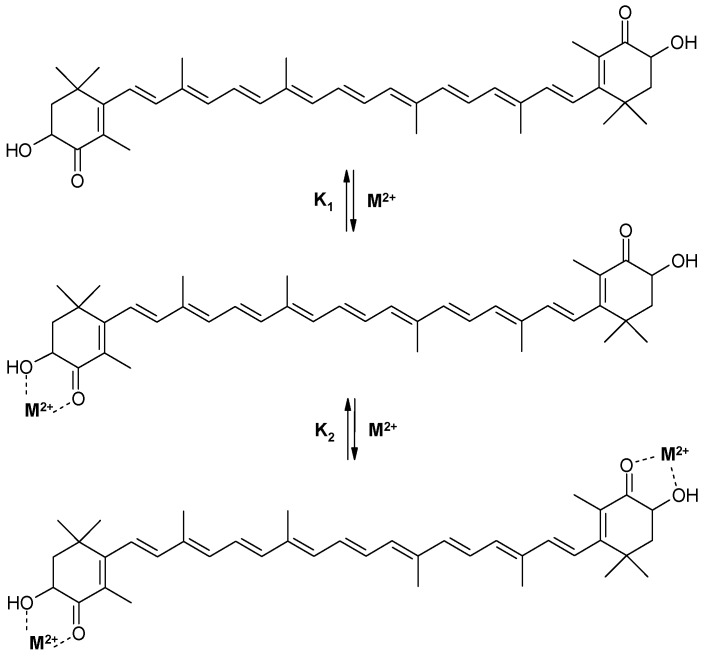
Complexation of astaxanthin with divalent metal ions.

We have found [12] that astaxanthin forms stable chelate complexes with metal ions Fe^2+^, Zn^2+^, and Ca^2+^ which produce significant changes in astaxanthin’s absorption spectrum. The astaxanthin absorption maximum shifts in the presence of Fe^2+^ from 480 nm (pure astaxanthin) to 492 nm (chelate complex) simultaneously with the appearance of a shoulder at 520–700 nm. Similar changes were detected for the Ca^2+^ and Zn^2+^ complexes making them better filters of visible light than astaxanthin itself. Stability constants of astaxanthin chelate complexes with different divalent metal ions have been measured optically in ethanol solution. It was found [12] that both 1:1 and 2:1 chelate complexes (metal: astaxanthin) are formed in two steps with different equilibrium constants, K_1_ and K_2_ (Scheme 1). Values for stability constants for all metal complexes are presented in Table 1, the stability constants K_1_ for complexes with 1:1 stoichiometry being larger than the stability constants K_2_ for complexes with 2:1 stoichiometry. It was found that at low salt concentrations (<0.2 mM) in an ethanol solution, the complex of astaxanthin with metal ions exists mainly with 1:1 stoichiometry. At high concentration of salt (>0.2 mM) a 2:1 metal to astaxanthin complex is formed.

A theoretical study [11] supports our findings. Density functional theory (DFT) calculations have confirmed astaxanthin’s ability to form metal ion complexes with metal ions such as Ca^2+^, Cu^2+^, Pb^2+^, Zn^2+^, Cd^2+^ and Hg^2+^ and the presence of metal ions induces changes in the maximum absorption bands which are red shifted in all cases. Moreover, the antiradical capacity of some astaxanthin metal complexes was studied and it was found that chelate complexes are slightly better electron donors and better electron acceptors than astaxanthin.

### 2.2. The Ability of Astaxanthin to Esterify and Inability to Aggregate in the Ester Form 

The molecular structure of astaxanthin with hydroxyl (–OH) group(s) at position(s) C3(C3’) also allows its esterification to monoesters and diesters for a better stabilization of the free form, as found in nature. Astaxanthin forms monoesters and diesters with fatty acids. The formation of esters may have additional benefits since it prevents carotenoid aggregation (dimers formation). It is known [13] that aggregation is a natural process for xanthophylls which results in a significant increase in the yield of reactive triplet state under irradiation. We have shown that the ester formation completely prevents aggregation [14]. The two ester ends in a diester of astaxanthin would prevent a metal ion binding. However, a monoester of astaxanthin would be able to chelate at one end.

### 2.3. ROS Scavenging Ability of Astaxanthin. The High Oxidation Potential

Carotenoids are known antioxidants. They can interact with free radicals (R^•^) in three main ways, namely addition, hydrogen abstraction, and electron transfer [15]. The antioxidant activity is dependent on the carotenoid structure but also on the nature of the oxidizing radical species.

▪ Radical addition to the polyene chain
  R^•^ + Car → (Car–R)^•^
▪ Hydrogen Abstraction from the Car
  R^•^ + Car → (Car–H)^•^ + RH
▪ Electron Transfer Reaction
  R^•^ + Car → R^−^ + Car^•+^

Electrochemical studies (cyclic voltammetry) of carotenoids in solution [16,17] show that upon electron transfer from the carotenoid molecule, the radical cation Car^•+^ is formed at an oxidation potential $E_{1}^{0}$. A second lost electron forms the dication Car^2+^ (Equation (2), Scheme 2) at an oxidation potential $E_{2}^{0}$ A neutral radical #Car^•^ forms at a reduction potential $E_{3}^{0}$ by reducing the cation #Car^+^ (Equation (3)) (this cation being formed upon proton loss from Car^2+^ in Equation (5)). 

A carotenoid neutral radical #Car^•^ can also form upon proton loss from Car^•+^ (Equation (6)) and thus we can call it a proton loss neutral radical to emphasize deprotonation of Car^•+^. This proton loss neutral radical was detected not just in solution, but in siliceous solid matrices (see Section 2.4.3) and, surprisingly, in vivo (Section 2.4.1).

Astaxanthin (0.768 V) has a higher oxidation potential compared to other carotenoids such as β-carotene (0.634 V), zeaxanthin (0.63 V) and lycopene (0.60 V) [16,17,18,19]. The position and the more polar nature of astaxanthin’s substituents compared to other carotenoids may explain its higher antioxidant activity. Table 2 lists the first oxidation potentials ($E_{1}^{0}$) of selected carotenoids determined in solution, using cyclic voltammetry. These values have been standardized to the potential of ferrocene of 0.528 V vs. saturated calomel electrode (SCE) given in a previous study [19]. The second oxidation potentials ($E_{2}^{0}$) for formation of Car^2+^ and the reduction potentials $E_{3}^{0}$ for formation of carotenoid neutral radicals #Car^•^ are not listed here. The first oxidation potential of astaxanthin determined versus SCE is 0.768 V, while the first oxidation potentials for the monoester and diester of astaxanthin versus SCE are slightly higher, 0.774 V and 0.775 V, respectively [18].

**Figure 2:**
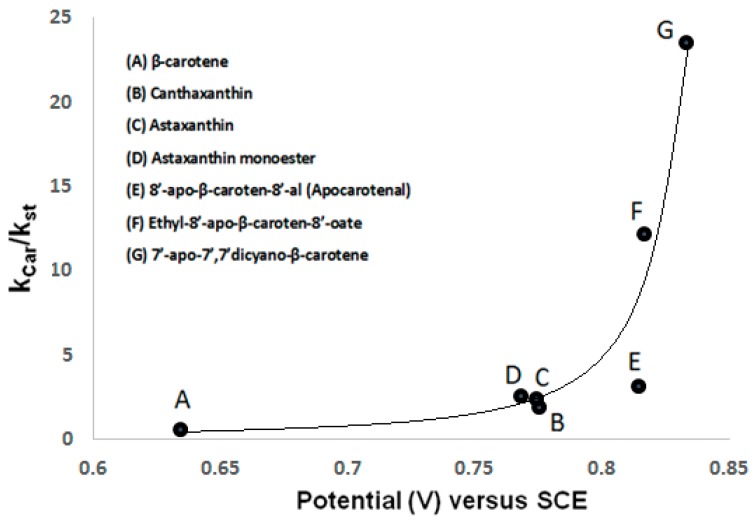
Plot of first oxidation potential versus scavenging ability (K_car_/K_st_) of carotenoids A–G. K_car_ reflects all possible ways of radical interaction with carotenoids and K_st_ is the relative spin trapping rate.

We have shown [18] that the first oxidation potential increases from 0.634 V (versus SCE) for β-carotene with no accepting electron groups to 0.775 V for canthaxanthin and 0.768 V for astaxanthin, both with electron accepting groups (Table 2). Using electrochemical measurements in combination with EPR spin trapping studies, we have found that with increasing first oxidation potential of the carotenoid molecule, the relative scavenging ability (K_car_/K_st_) towards peroxyl radicals ^•^OOH radicals formed in a Fenton reaction increases [20]. Determined values of K_car_/K_st_ [20] are listed in Table 2 and were used to show the direct relationship between the first oxidation potential and the scavenging ability of carotenoids (Figure 2). Figure 2 shows the higher antioxidant ability of astaxanthin when compared to a carotenoid with low oxidation potential such as β-carotene. It is also shown that the scavenging ability of the ester carotenoid ethyl-8′-apo-β-caroten-8′-oate (0.816 V) and that of a carotenoid containing cyano groups, 7′-apo-7′,7′-dicyano-β-carotene (0.833 V), increases by a factor of 4 and by a factor of 8, respectively, versus that of the aldehyde 8′-apo-β-caroten-8′-al (0.814 V). The ability of carotenoids to scavenge peroxyl radicals ^•^OOH increases significantly with an increase in the redox potential [20].

**Figure 3:**
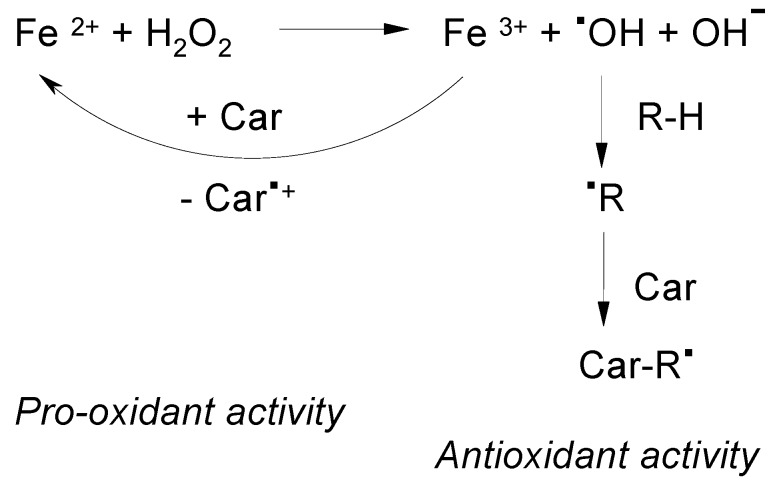
The suggested mechanism of the antioxidant and pro-oxidant activity of carotenoids in the presence of Fe ions [21].

Carotenoids can increase or decrease the total yield of free radicals produced in a Fenton reaction [21] depending on their oxidation potential and the nature of the free radical (Figure 3). Figure 3 presents the Brutto formula of the Fenton reaction used in [21] to illustrate the mechanism of ^•^OOH radical formation in excess of hydrogen peroxide H_2_O_2_ [21]. According to the modern point of view [22] the intermediates of the Fenton reaction are high valent ferryl species. At present, the formation of the ^•^OH hydroxyl radical in this reaction is still under discussion. In favor of the ^•^OH radical formation are the results of more recent EPR spin trapping experiments using the OH selective TMIO (2,2,4-trimethyl-2*H*-imidazole 1-oxide) spin trap in the absence and in the presence of dimethyl sulfoxide (DMSO) [9]. Possible reactions, rate constants and the time course during the Fenton reaction were provided in reference 20, the process being monitored optically. In a carotenoid-driven Fenton reaction [21] a carotenoid with a low oxidation potential can have a pro-oxidant activity increasing the total radical yield (K_Car_/K_st_ << 1). Because of its lower oxidation potential β-carotene has the ability to reduce Fe^3+^ more easily. Astaxanthin, with the higher oxidation potential acts as an antioxidant decreasing the total radical yield (K_Car_/K_st_ > 1).

It was determined [12] that the oxidation potential of astaxanthin decreases in the presence of salts lowering its ability to scavenge radicals and decreasing its relative antioxidant ability. In the presence of salts, the stability of astaxanthin radical cations Ast^•^^+^ and dications Ast^2^^+^ decreases and there is an increase in the lifetime of the neutral radicals #Ast^•^ formed by proton loss from the radical cation Ast. An electrochemical study [12] showed significant shifts in the oxidation and reduction potentials of astaxanthin and a decrease in the stability of Ast^•^^+^ with enhanced proton loss neutral radical formation in metal complexes of astaxanthin.

We have shown above that depending on their oxidation potential, carotenoid molecules can scavenge toxic free radicals. The ability to lose terminal protons from the radical cation is also dependent on the oxidation potential of the carotenoid molecule: the higher the oxidation potential of the carotenoid molecule, the lower the acidity of the terminal proton. For example, the pKa for zeaxanthin radical cation Zea^•^^+^ is 4 but near neutral 7 for astaxanthin radical cation Ast^•^^+^, indicating that Zea^•^^+^ is a stronger acid (more readily loses a proton) than Ast^•^^+^. This implies that proton donation to a neighboring acceptor molecule decreases for Ast^•^^+^ versus Zea^•^^+^. However, in a chelate complex with metal ions, deprotonation rate of the astaxanthin radical cation Ast^•^^+^ will increase considerably.

### 2.4. The Ability of Carotenoids to Form Proton Loss Neutral Radicals under High Illumination

#### 2.4.1. In *Arabidopsis thaliana* Plant 

**Figure 4:**
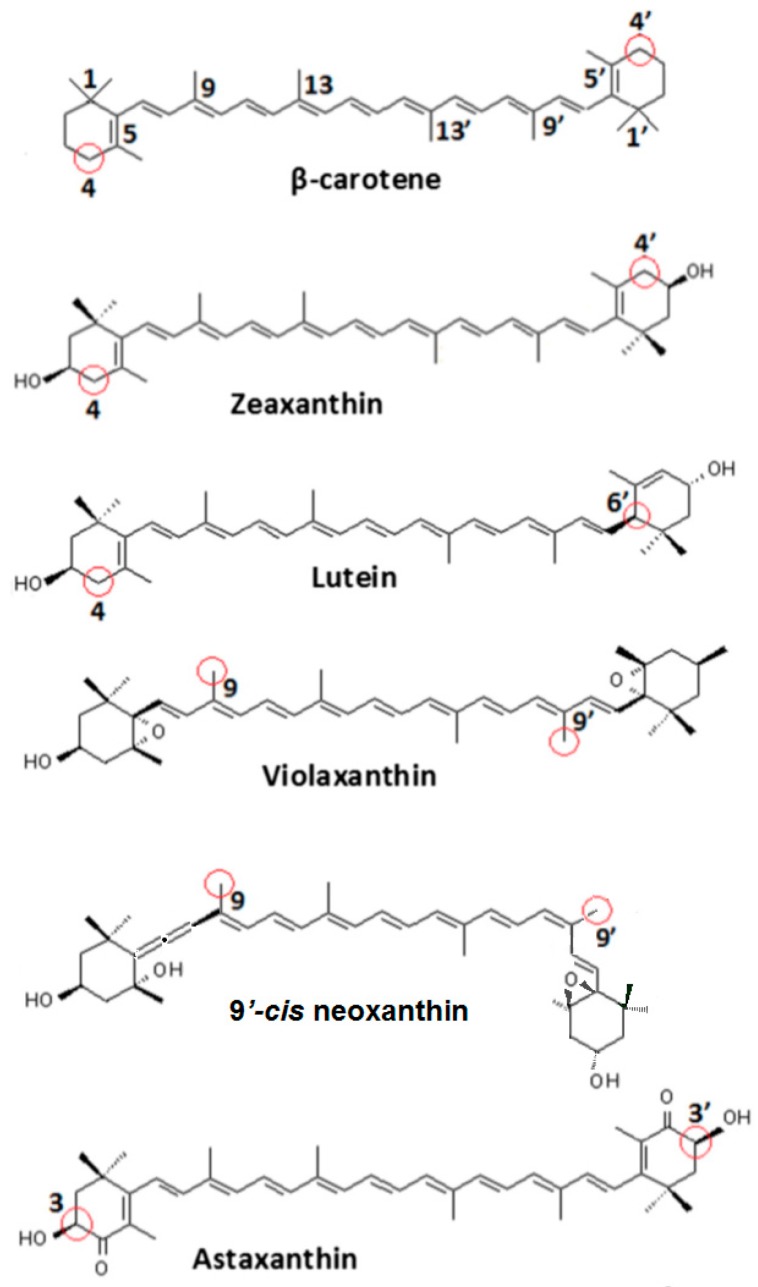
Carotenoid structures. Circled in red are the most favorable positions for proton loss from a radical cation.

Carotenoid radicals such as radical cations (Car^•^^+^) formed by electron transfer from carotenoid molecules (see Figure 4) and proton loss neutral radicals #Car^•^ formed under high light intensity by deprotonation of Car^•^^+^ at the most favorable position have been found to occur not only in solution, but in photosynthetic media. The proton loss neutral radical, first observed to occur in irradiated PSII samples as #β-Car^•^ (beta-carotene neutral radical) [23] and then in the *Arabidopsis thaliana* plant [24] as #Zea^•^ (zeaxanthin neutral radical), has been suggested [25,26] to provide additional photo protection of plants to that provided by Car^•^^+^. Since the radical cation Zea^•^^+^ is a weak acid (with pK_a_ 4–7), loss of a proton will occur to a water acceptor and the proton loss neutral radical, #Zea^•^, is formed. The presence of a neutral radical in the vicinity of an excited Chl molecule can cause the quenching of the excited state of Chl by J exchange [27]. Quenching of fluorescence by J exchange for either an excited single or triplet state has been accomplished by attaching a stable nitroxide neutral radical as far away as 9 Å from a fluorescing molecule. This was demonstrated by Hideg [27] via fluorescence quenching in two reactive oxygen species sensors; singlet oxygen specific DanePy and OH-1889NH, which reacts with both singlet oxygen and superoxide radicals. A similar situation would occur if #Zea^•^ is near an excited Chl in light harvesting complex II (LHCII). Each #Zea^•^ would become a potent free radical trap for large numbers of excited Chl, potentially making a major contribution to qE even if its quantum yield is small. This supports the idea of a possible additional mechanism for quenching Chl’s excess energy in which the longer lived proton loss neutral radical of the carotenoid plays the role of the quencher [25]. 

Results [28,29,30,31] have shown that zeaxanthin participates in non-photochemical quenching in plants, specifically qE, by forming a charge transfer complex Zea^•^^+^…Chl^•−^ (150 ps lifetime) which separates into the radical cation Zea^•^^+^ and the radical anion Chl^•−^. Transient generation of Zea^•^^+^ (~11 ps) allows quenching the excess energy of Chl excited states [28]. Energy is transferred from chlorophyll molecules to the charge transfer complex which then undergoes charge separation into Zea^•^^+^ and Chl^•−^. The weak acidity of zeaxanthin radical cation enables proton loss from a fraction of Zea^•^^+^ to preassembled water acceptors to form the proton loss neutral radical, #Zea^•^. The structure of pea light harvesting complex II (LHC II) at 2.5 Å resolution (Protein Data Bank 2BHW) shows available water molecules within a few to several Å of the zeaxanthin’s cyclohexene ring [32]. Similarly, crystal structure of the CP29 minor antenna component (Protein Data Bank 3PL9) also shows an axial water ligand in close proximity (<4 Å) to the terminal ring (C4) of zeaxanthin [24]. Proton loss from Zea^•^^+^ to preassembled water acceptors would leave #Zea^•^ and Chl^•−^ separated in the protein and prevent recombination of charges because deprotonation drastically shifts the redox potential of Zea. Proton transfer reactions can occur in tens of fs to a few ps [33,34,35,36] making deprotonation feasible during the 150 ps lifetime of the charge transfer complex. The radical anions BChl^•−^ are known to migrate through the lattice undergoing facile electron transfer with other BChls in the protein complex [37,38,39]. During the charge separation, both #Zea^•^ and Zea^•^^+^ can encounter and quench Chl excited states. To reform the charge transfer complex and return to the molecular ground state, the cation Zea^•^^+^ and anion Chl^•−^ need to come together again [25].

**Figure 5:**
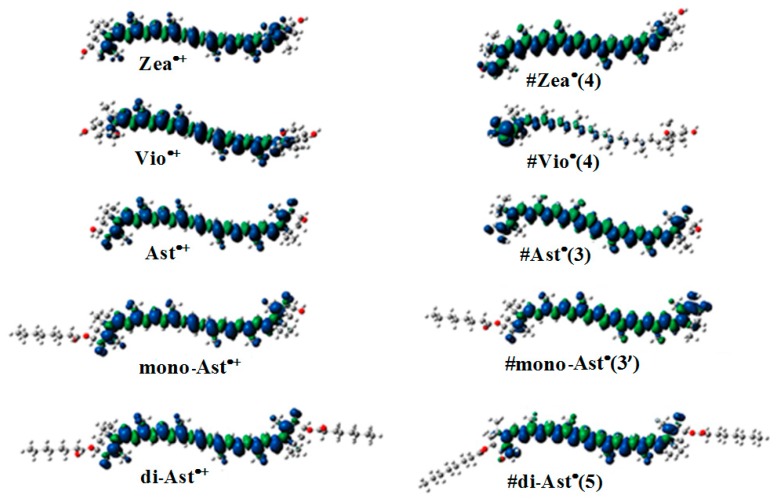
Unpaired spin density distribution for radical cations (left) and the corresponding most favorable (lowest energy) proton loss neutral radicals (right) for selected carotenoids.

The most favorable location for proton loss from the radical cation to form a neutral radical occurs at the cyclohexene ends of the carotenoids. This proton loss extends the conjugation length providing additional stability and longer lifetime for the quenching neutral radical. Proton loss for Zea^•^^+^ to form #Zea^•^ is most favorable at C4 (or C4’ by symmetry) generating the proton loss neutral radical #Zea^•^ (4) (or #Zea^•^ (4’), where the position for proton loss is indicated in parenthesis. The unpaired spin density distributions of Zea^•^^+^ radical cation and that of most stable neutral radical #Zea^•^ (4) is shown in Figure 5, along with those for other carotenoids such as violaxanthin, astaxanthin, and its monoester and diester, for comparison. Notice in the case of violaxanthin neutral radical #Vio^•^ (4) that its unpaired spin density is localized on the C4, C5 and C6 atoms. The epoxide group at positions C5–C6 raises the energy barrier for proton loss at the cyclohexene end making proton loss unfavorable. 

In the case of astaxanthin, the effect of hydroxyl (–OH) and keto (C=O) groups at position(s) C3(C3’) and C4(C4’) is to reduce the reactivity of this molecule by preventing hydrogen abstraction from these positions. DFT calculations have shown [40] that proton loss at C3(C3’) position of the radical cation and proton migration from hydroxyl group to the keto group generates the most favorable neutral radical #Ast^•^ (3) (or #Ast^•^ (3’) by symmetry) (Figure 5). For astaxanthin monoester proton loss at C3’ at the ester-free end and proton migration from hydroxyl group to the carbonyl group generates the lowest energy neutral radical #mono-Ast^•^ (3’). Proton loss at these positions is prevented in the diester by the two ester groups and thus proton loss is most favorable at the C5(C5’) position(s) instead (Figure 5).

#### 2.4.2. In Light Harvesting Complex II (LHCII)

**Figure 6:**
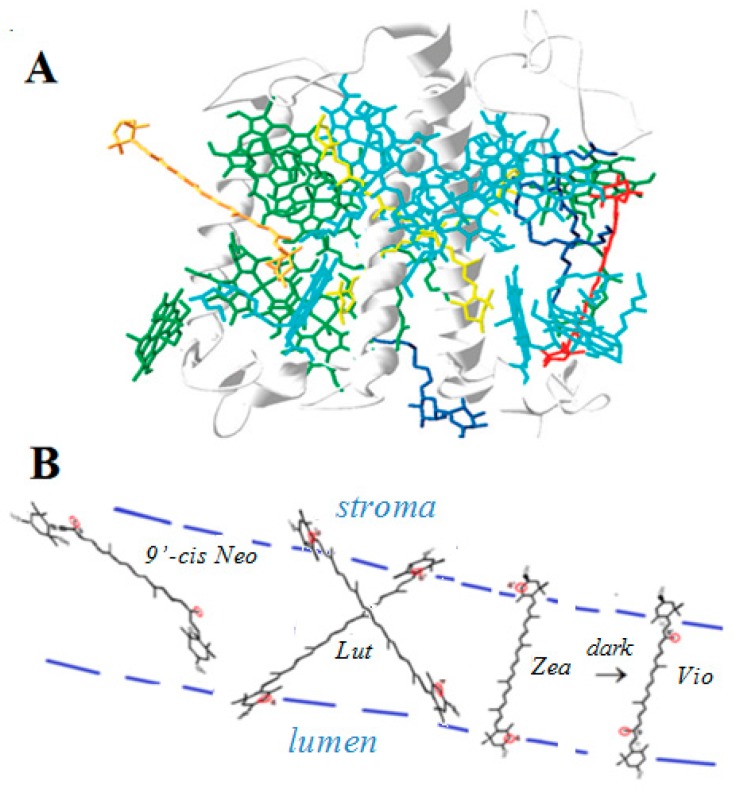
(**A**) LHCII [32] showing the location of the four carotenoids: zeaxanthin (under light) or violaxanthin (under dark) in red, two lutein molecules in a cross position in yellow, and 9’-*cis* neoxanthin in orange. (**B**) Schematic diagram of the location of the four carotenoids in LHCII indicating the most favorable proton loss in red. Proton loss can occur off the ends of the radical cations of zeaxanthin (Zea) and lutein (Lut), also enabled by the nearby aqueous lumen and stroma. Proton loss off the ends of the radical cations of violaxanthin (Vio) or 9’-*cis* neoxanthin (9’-*cis* Neo) is prevented by their structures and by embedment in the hydrophobic region.

A correlation between the quenching ability of the four carotenoids lutein, zeaxanthin, violaxanthin and 9’-*cis* neoxanthin present in LHCII (Figure 6) and their ability to form proton loss neutral radicals was found [41]. Zeaxanthin and lutein radical cations known as quenchers in photobiological studies formed neutral radicals on our siliceous solid supports, while violaxanthin and 9’-*cis* neoxanthin, that are nonquenchers, did not form proton loss neutral radicals on solid supports [41,42,43]. Interestingly, the location of zeaxanthin and lutein in LHCII, near the aqueous proton-accepting lumenal and stromal regions, is also convenient for proton loss so that a radical cation would easily form a proton loss neutral radical (Figure 6). Zeaxanthin and lutein radical cations can thus loose a proton to the lumenal and stromal regions of the thylakoid membrane while violaxanthin and 9’-*cis* neoxanthin cannot because their structures contain epoxy group(s) and the allene bond that prevent proton loss at the cyclohexene ends. 

#### 2.4.3. In Solid Supports: Silica-Alumina, Silica Gel, MCM-41, Metal-Substituted MCM-41 and TiO_2_

In plants, proton loss neutral radicals formed by radical cation deprotonation are short-lived (~100 ps) and difficult to detect. In vivo proton loss was detected at the cyclohexene ends only [24], as a result of the environment around the carotenoid, like in an organized assembly. However, in an unorganized assembly, like a siliceous solid matrix, proton loss occurs at all positions and all proton loss neutral radicals are stabilized. In a solid matrix proton loss occurs not only from the cyclohexene ends but also from less energetically favorable positions such as the methyl groups attached to the polyene chain. As an alternative for the study of their properties, carotenoid radicals, both Car^•^^+^ and #Car^•^, were long-term stabilized on solid supports such as silica-alumina, silica gel, and molecular sieves like MCM-41 and metal-substituted MCM-41 where they are stable for as long as hours to days [25]. Published EPR literature has shown that the yield of these radicals (Car^•^^+^ and #Car^•^) formed on such solid supports is largely increased during light irradiation and in the MCM-41’s molecular framework. When carotenoids are adsorbed on MCM-41 or metal-substituted Cu-MCM-41 [44], Ti-MCM-41 [12], Al-MCM-41, Ni-MCM-41 [45] and Fe-MCM-41 [46], Car^•^^+^ is initially formed by electron transfer to the matrix. Upon light irradiation of these samples, #Car^•^ is formed by Car^•^^+^ deprotonation, providing an order of magnitude increase in the concentration of radicals. Also, the addition of a metal to the MCM-41 matrix provides a larger increase in photo yield when compared to the MCM-41 matrix lacking the metal [44].

Because of the possible role of these neutral radicals in providing a secondary photo protection pathway in addition to the known photo-protection by Car^•^^+^, it is important to understand the mechanism of their formation in different matrices and the conditions in which they occur. Also, what is the role of metals enhancing the likelihood of #Car^•^ formation? The ability of metal ions to produce ROS under light irradiation and to cause oxidative stress in living organisms is well known [47,48,49]. Reviewing and linking the information from previous publications helped us to better understand it. We need to consider possible reactions taking place in a matrix that lead to the formation of the superoxide radical anion O${}_{2}^{•-}$. We suggest that the superoxide radical anion plays a role in proton abstraction from the carotenoid radical cation to generate a proton loss neutral radical.

##### Mechanism of Proton Loss Neutral Radical Formation in an Irradiated TiO_2_ Matrix. Sources of O${}_{2}^{•-}$

A study [50] shows that when a TiO_2_ matrix is irradiated, electrons are trapped on the surface and holes are created. A broad EPR spectrum at g = 1.99 was assigned to the photo-generated electrons trapped as Ti^3+^ surface ions ((e${}_{tr}^{-}$)Ti^3+^) according to the equation TiO_2_ + *hν* → (e${}_{tr}^{-}$)Ti^3+^ + h^+^. A second study [51] showed that on an irradiated TiO_2_ matrix, the O${}_{2}^{•-}$ species was generated, confirmed by using the EPR spin trapping technique. It was assumed that O${}_{2}^{•-}$ formation on irradiated TiO_2_ may occur as a result of both hole and electron trapping reactions. Photo-generated holes on TiO_2_ colloids could be trapped at the lattice oxide ion sites as the O${}_{2}^{•-}$ species according to reaction: O${}_{2}^{2-}$ + h^+^ → O${}_{2}^{•-}$. Previous pairwise trapping of holes at diamagnetic O${}_{2}^{2-}$ ions has been proposed for MgO [52]. MgO crystals which contained dissolved traces of H_2_O eventually acquired an excess oxygen content because OH^−^, associated with cation vacancies converted into peroxy anions, O${}_{2}^{2-}$, and into molecular H_2_. H_2_ was lost from the crystal but the vacancy-bound O${}_{2}^{2-}$ remained at self-trapped positive holes resulting in a negatively charged surface. Adsorbed O${}_{2}^{2-}$ ions in MgO also produced O${}_{2}^{•-}$ via reaction with ^•^OH (O${}_{2}^{2-}$ + ^•^OH → O${}_{2}^{•-}$+ OH^−^) [52]. Superoxide radical anion O${}_{2}^{•-}$ formation can also proceed through the reduction of molecular oxygen by the photo-generated electrons (e${}_{tr}^{-}$ + O_2_ → O${}_{2}^{•-}$) [51].

The EPR spin trapping technique used in the study of TiO_2_ colloids [51] revealed not only the features from O${}_{2}^{•-}$ but also from the perhydroxyl radical ^•^OOH. In the presence of water or OH groups in the system, the perhydroxyl radical formation has been reported. In air-saturated organic solvents containing water, the superoxide radical anion O${}_{2}^{•-}$ can form perhydroxyl radicals according to the reaction: O${}_{2}^{•-}$ + H^+^ → ^•^OOH [53,54] that are stronger oxidants than O${}_{2}^{•-}$. It has been also reported [55,56] that under UV illumination of the TiO_2_/O_2_ system, O_2_ can react with Ti^3+^-OH surface sites to make ^•^OOH . In the absence of other reactants, the superoxide radical anion O${}_{2}^{•-}$ decays via a second-order reaction producing hydrogen peroxide H_2_O_2_ and singlet oxygen ^1^O_2_ [53,54].

H_2_O_2_ has been detected as a product of irradiated TiO_2_ in aqueous dispersions [57,58] and photo-degradation of different dyes in the presence of TiO_2_. When TiO_2_ suspensions in CH_2_Cl_2_ were irradiated at 546 nm in the presence of the spin trap 4-oxo-TMP, enhanced detectable nitroxide signals were observed in carotenoid concentration ranging from 10^−5^ to 10^−4^ M. At concentrations higher than 10^−4^ M, carotenoids decreased the enhancement effect and the signal was eliminated by 1.5 × 10^−3^ M β-carotene. This was attributed to the quenching of ^1^O_2_ by carotenoids [51].

##### How do Proton Loss Neutral Radicals Form in a Siliceous MCM-41 Matrix Containing a Metal-Oxygen (M-O) Bond (M=Al, Fe, Ti)?

By considering the reactions above, we can explain the formation of a neutral radical upon light exposure when Car is adsorbed on a matrix containing a metal-oxygen (M-O) bond such as the metal-substituted MCM-41 supports (Ti-MCM-41, Cu-MCM-41, Al-MCM-41, Fe-MCM-41) [12,44,45,46]. EPR measurements show that Car^•^^+^ is formed in the absence of light by electron transfer to the Lewis acid sites of the matrix. Upon light illumination when the Car^•^^+…^M-O(e${}_{tr}^{-}$) is formed, and the O${}_{2}^{2-}$ in metal oxide systems allows for the formation of O${}_{2}^{•-}$ by reaction with the trapped electron e${}_{tr}^{-}$ at the metal-oxygen bond, O${}_{2}^{•-}$ can abstract a proton form Car^•^^+^ to form #Car^•^.

In the presence of adsorbed O_2_, light activation can form O${}_{2}^{•-}$ by reaction with the trapped electron e${}_{tr}^{-}$ at the metal-oxygen bond (M-O(e${}_{tr}^{-}$)) and Car^•^^+^. Since the samples were prepared in evacuated EPR tubes one can argue that formation of #Car^•^ from Car^•^^+^ on metal-substituted MCM-41 matrices has not relied on the presence of surface O_2_. Instead, the M-O bond would need to provide a source of O${}_{2}^{2-}$ ions, so that upon light activation of the adsorbed carotenoid, trapped electrons would occur forming O${}_{2}^{•-}$. However, the absence of surface oxygen, despite all efforts to eliminate has been shown to not be possible (no matter the vacuum applied) [45].

The electron transfer of β-carotene, canthaxanthin and 7’-apo-7’,7’-dicyano-β-carotene embedded in MCM-41 and Ti-MCM-41 was studied as a function of oxidation potential of the carotenoid [59]. β-carotene possessing the lowest oxidation potential gave the highest radical cation photo yield on MCM-41 lacking the metal. A broad EPR signal for irradiated β-carotene at 77 K was shown to be due the overlap of Car^•+^ and O${}_{2}^{•-}$ signals. The O${}_{2}^{•-}$ species was identified by the very characteristic EPR pattern of g = 2.0115, g = 2.029 and g = 2.000. It is known [45,60] that photo-irradiation of MCM-41 produces O${}_{2}^{•-}$. Further photo-irradiation at 77 K resulted in a decrease in the Car^•^^+^ signal due to the formation of the dication Car^2+^ produced by the electron transfer form Car^•^^+^ to the MCM-41 matrix according to β-Car^•^^+^ − e^−^ → β-Car^2+^. When β-carotene was imbedded in Ti-MCM-41, two different species were formed Ti^4+^(O${}_{2}^{•-}$) and Ti^3+^ produced by hydrogen reduction. This species is formed by cleavage of one Ti-O bond forming the Ti^3+^ species after complete consumption of O_2_. Complete removal of O_2_ from the samples is difficult [45,61].

To study their properties, the astaxanthin radical cation (Ast^•^^+^) and the astaxanthin neutral radicals #Ast^•^ were long-term stabilized (for as long as hours to days) on solid supports such as silica-alumina, MCM-41 and metal-substituted MCM-41 and studied with EPR techniques [12,40]. In the absence of light, astaxanthin in a methylene chloride solution adsorbed on silica-alumina produced only Ast^•^^+^. Light irradiation produced both Ast^•^^+^ and #Ast^•^ on silica-alumina and in siliceous MCM-41. An order of magnitude increase in the radicals concentration was observed when astaxanthin was adsorbed on Ti-MCM-41 [12] in the presence of light versus when adsorbed on MCM-41 indicating that Ti^4+^ enhances the radical yield. This behavior was also observed for other carotenoids adsorbed on MCM-41 containing other metals such as Al-MCM-41, Fe-MCM-41 or Cu-MCM-41, the metal presence and light irradiation producing an increase in carotenoid radical concentration [44,45,46]. 

## 3. Discussion

So why is astaxanthin so successful when exposed to stressful conditions? How do free astaxanthin (10%), the diester of astaxanthin (15%) and the monoester of astaxanthin (70%) present in *Haemetococcus pluvialis* algae provide such great photoprotection under stressful conditions such as high light, high salinity and ROS? 

First, the chelating of metal ions will result in increasing acidity of the astaxanthin radical cation and accelerate its deprotonation, following more efficient quenching of Chl excited state by the proton loss neutral radicals of astaxanthin. According to literature data, chelation can increase deprotonation rate by several orders of magnitude [62,63]. Deprotonation will take place during the short timescale (hundreds of ps), so the xanthophyll cycle continues to operate on the long term (ms or longer). This is important to avoid irreversible reactions with diffusing O_2_ [64], so on the long timescale (min) the astaxanthin molecule can scavenge ROS.

**Scheme 3:**
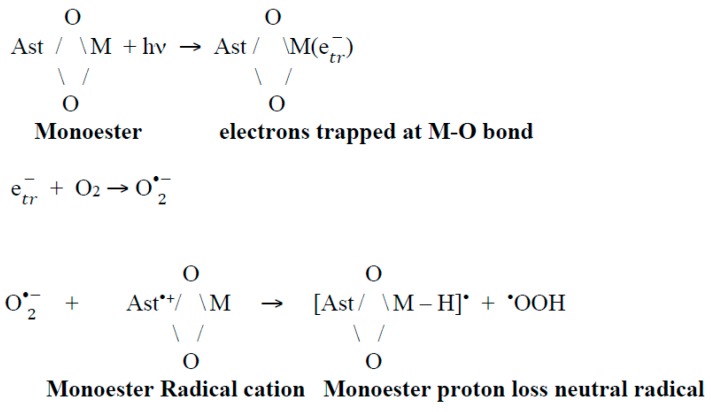
Suggested reactions for monoester.

We can propose that a second route of neutral radical of astaxanthin formation might be the reaction of the radical cation with the superoxide radical O${}_{2}^{•-}$. Different studies [59,65,66] demonstrate the reason for the large increase in neutral radicals: that is the abstraction of protons at the ends of the structure (proton at C3 and C3’ position) from the Ast^•^^+^ radical cation or even the astaxanthin molecule by the reaction with the O${}_{2}^{•-}$ generated by light irradiation. These results are critical for the photo protection of astaxanthin in salty ponds. For example, the presence of the astaxanthin monoester and light produces trapped electrons at the metal-oxygen bond that can react with surface O_2_ to produce O${}_{2}^{•-}$. This radical can abstract a proton from the carotenoid radical cation to generate a quenching proton loss neutral radical (Scheme 3).

Based on the above-mentioned studies, deprotonation of the radical cations under high light leads to formation of proton loss neutral radicals for all three astaxanthin species (free astaxanthin, monoester and diester) [40]. Their neutral radicals formed under high light intensity can be very efficient quenchers of excited singlet and triplet states of chlorophyll. Neutral radicals quench the excited states of chlorophyll in a secondary photoprotection pathway [24,25]. Astaxanthin and its monoester can also trap metal ions in the open ponds forming metal complexes with large stability constants [12]. The stability constants and stoichiometry of such complexes were found to be solvent and salt sensitive. The stability constant K_1_ when astaxanthin coordinates with a metal ion at one end is much larger than K_2_ when it coordinates at both ends. The monoester coordinating with a metal ion on one end would be stable, similar to free astaxanthin coordinating with a metal. While astaxanthin and its monoester can coordinate with a metal at one end, a diester would not be able to coordinate with metals but it would still form proton loss neutral radicals available for quenching Chl excited states. The formation of proton loss neutral radicals of astaxanthin, its esters and metal complexes was confirmed by electrochemical studies [12,18].

## 4. Conclusions

In conclusion, the efficient protective role of astaxanthin in *H. pluvialis* in response to various environmental stress conditions such as high light, ROS or salt stress is due to astaxanthin’s structural features which determine its unique properties: the ability to form chelate complexes with metals, its large oxidation potential, the inability to aggregate in the ester form, and the ability to form proton loss neutral radicals under high illumination and in the presence of metal ions. The formation of neutral radicals occurs on a picosecond time scale, and these radicals can exist for microseconds-long enough time to play the role of a quencher of Chl excited states and then relax back to form neutral molecules. We can propose two possible mechanisms of proton loss by the astaxanthin radical cation, namely deprotonation in chelate complexes and the reaction with the superoxide radical. Anyway, independent of the mechanism of its formation, if neutral radicals of astaxanthin are formed in *H. pluvialis* under high irradiation and in the presence of metals, they would be very effective quenchers of the chlorophyll excited states. In addition to quenching of chlorophyll excited states, the antioxidant features of astaxanthin are determined by its ability to inhibit ROS formation by several mechanisms: (a) direct trapping of ROS with highest efficacy; (b) trapping h^+^ holes produced by metal ions under irradiation; (c) chelating transition metal ions thus preventing their participation in Fenton, photo-Fenton and Haber-Weiss reactions.

## Figures and Tables

**Scheme 2:** Reaction mechanism of carotenoids. Car: carotenoid molecule, Car^•+^: carotenoid radical cation, Car^2+^: carotenoid dication, #Car^+^: deprotonated carotenoid cation (# indicates proton loss), #Car^•^: proton loss neutral radical (# indicates proton loss).

$$\begin{array}{l} \text{Heterogeneous electrode reactions:}\\ \mathrm{Car}\overset{E_{1}^{0}}{⇌}{\mathrm{Car}}^{•+}+e^{-}\;(1)\\ {\mathrm{Car}}^{•+}\overset{E_{2}^{0}}{⇌}{\mathrm{Car}}^{2+}+e^{-}\;(2)\\ \#{\mathrm{Car}}^{+}+e^{-}\overset{E_{3}^{0}}{⇌}\#{\mathrm{Car}}^{•}\;(3)\\ \text{Homogeneous electrode reactions:}\\ {\mathrm{Car}}^{2+}+\mathrm{Car}\overset{K_{\mathrm{com}}}{⇌}2{\mathrm{Car}}^{•+}\;(4)\\ {\mathrm{Car}}^{2+}\overset{K_{\mathrm{dp}}}{⇌}\#{\mathrm{Car}}^{+}+H^{+}\;(5)\\ {\mathrm{Car}}^{•+}\overset{K^{\prime }{}_{\mathrm{dp}}}{⇌}\#{\mathrm{Car}}^{•}+H^{+}\;(6)\\ \text{Side reactions}:\\ {\mathrm{Car}}^{•+}\to \mathrm{Unknown}\mathrm{Products}\;(7)\\ {\mathrm{Car}}^{2+}\to \mathrm{Unknown}\mathrm{Products}\;(8)\\ \#{\mathrm{Car}}^{+}\to \mathrm{Unknown}\mathrm{Products}\;(9)\\ \#{\mathrm{Car}}^{•}\to \mathrm{Unknown}\mathrm{Products}\;(10) \end{array}$$

**Table 1:** Stability constants of astaxanthin chelate complexes with divalent metal ions in ethanol solution, adopted from reference [12].

Salt	Ca(ClO_4_)_2_	Zn(ClO_4_)_2_	Fe(ClO_4_)_2_
Stability constants	K_1_ = 23,000 M^−1^ K_2_ = 5000 M^−1^	K_1_ = 3000 M^−1^ K_2_ = 1300 M^−1^	K_1_ = 3000 M^−1^

**Table 2:** First oxidation potentials ($E_{1}^{0}$) for selected carotenoids and the relative scavenging ability (K_car_/K_st_) towards peroxyl radicals.

Carotenoid	$E_{1}^{0}$ vs. SCE	K_car_/K_st_ [20]
β-carotene [19]	0.634 ± 0.001	0.64
canthaxanthin [19]	0.775 ± 0.001	1.96
astaxanthin [18]	0.768 ± 0.001	2.60
astaxanthin monoester [18]	0.774 ± 0.001	2.50
astaxanthin diester [18]	0.775 ± 0.001	not measured
8′-apo-β-carotene-8′-al [16]	0.814 ± 0.005	3.22
ethyl-8′-apo-β-caroten-8′-oate [16]	0.816 ± 0.005	12.20
7′-apo-7′,7′-dicyano-β-carotene [16]	0.833 ± 0.005	23.60
